# Rethinking Obesity Prevention: A Call for Strategic and Operational Clarity

**DOI:** 10.1007/s13679-026-00708-5

**Published:** 2026-04-07

**Authors:** Ximena Ramos Salas, Brad Hussey, Angela S. Alberga, Ted Kyle, Francine Small

**Affiliations:** 1 Lund University, Lund, Sweden; 2 Replica Communications, Kristianstad, Sweden; 3 Bias180, Dundas, Canada; 4 Replica Communications, Hamilton, Canada; 5 Concordia University, Montreal, Canada; 6 ConscienHealth, Pittsburgh, USA; 7 Dawson College, Montreal, Canada

**Keywords:** Obesity, Obesity prevention, Chronic disease prevention, Health promotion

## Abstract

**Background:**

Despite decades of initiatives promoting individual behavior change, obesity rates remain high and continue to rise globally, indicating that what is commonly labelled as “obesity prevention” is not, in practice, preventing obesity. While health promotion strategies and policy interventions have demonstrated improvements in environments, health behaviors, and industry practices, their effects on obesity incidence and prevalence remain modest.

**Objective:**

To examine the mismatch between current obesity prevention practices and contemporary scientific understanding of obesity as a chronic disease, and to propose a reframed approach to prevention.

**Methods:**

Conceptual analysis informed by empirical evidence and field insights. The authors conceptualize a broad misalignment between scientific understanding of obesity and prevention practice.

**Results:**

Current prevention efforts primarily target individual health behaviors and environmental factors related to food and physical activity, rather than addressing the complex causes of obesity. Contemporary clinical guidance defines obesity as a chronic, complex, and relapsing disease characterised by excess or dysfunctional adiposity that impairs health, shaped by biological, genetic, behavioral, and environmental factors. Behavioral interventions produce small changes in BMI and do not reliably prevent the onset or progression of obesity. Evidence demonstrates that behaviors are partly driven by underlying biological and brain-based mechanisms, and that physiological systems resist sustained weight change. As a result, interventions focused primarily on individual behavior are insufficient to prevent a disease governed by interacting biological and systemic processes. Drawing on empirical evidence and field insights, the authors show that prevention efforts are further limited by narrow outcome measures, short-term policy cycles, fragmented accountability, and inconsistent disease classification. Obesity prevention efforts do not align with current scientific understanding of obesity as a chronic, complex, and relapsing disease. Obesity prevention should be redefined to explicitly target disease onset and progression. An integrated, multi-level framework is needed to align health promotion, prevention, and clinical care, addressing biological, behavioral, and system (social, physical, commercial) drivers simultaneously.

## Introduction

Despite decades of initiatives promoting individual behavior change, obesity rates remain high and continue to rise globally [1]. This reflects a pervasive cultural narrative that weight is purely about personal choices and behaviors, rather than a complex, biological chronic disease. In addition, policy and practice spheres have failed to fully integrate obesity care and accommodations into health systems (e.g., structures and services that shape how obesity prevention, treatment, and long-term management are delivered). Nor have they developed coordinated, multi-level interventions spanning individuals, families, communities, and public policy. Multi-level obesity prevention and management should in theory include health promotion strategies, disease prevention programs, and long-term treatment and management services.

Health promotion strategies address the social, economic, and environmental conditions that shape population health by creating the knowledge, skills, environments, and policies that support healthy choices, healthy living, and health equity [2]. The WHO Healthy Cities initiative, for example, is designed to help cities create healthy policies and environments by improving walkability, housing, transport, and social inclusion, making healthy choices easier and leading to higher physical activity, better services, and stronger community health systems [3].

Disease prevention strategies focus on people at elevated risk of developing chronic diseases, such as obesity, emphasizing early screening, intervention during key life stages, and programs that integrate biological, behavioral, and psychosocial support [4]. The National Diabetes Prevention Program, for example, helps people with prediabetes prevent type 2 diabetes through year-long behavioral coaching on healthy eating, physical activity, and behavior change strategies [5].

Treatment programs provide long-term care for those living with chronic diseases, including psychological and behavioral therapy, pharmacotherapy, and surgery when needed and appropriate, while also addressing stigma and access barriers [6]. For example, the Health Services Executive Specialist Weight Management Service is a publicly funded program in Ireland that treats adults with obesity through primary care physician referral and provides coordinated medical, dietetic, psychological, and behavioral support to improve long-term health [7].

## Shifting Away from Defining Obesity as a Matter of Personal Responsibility

To establish a foundation for integrated multi-level obesity prevention and management services, it is necessary to clarify how obesity is currently defined. Recent international guidance has reframed obesity as a chronic, complex, and relapsing disease influenced by biological, genetic and environmental influences rather than a simple consequence of poor lifestyle choices. The landmark 2020 Canadian Adult Obesity Clinical Practice Guideline (CPG) defines obesity as “a prevalent, complex, progressive and relapsing chronic disease, characterized by abnormal or excessive body fat (adiposity), that impairs health” [8]. Since 2020, many clinical practice guidelines and frameworks for obesity care have adhered to a similar definition (see: Irish and Chilean adaptations of the Canadian CPG [9, 10], and recent guidance published in/by Sweden [11], Netherlands [12], Mexico [13], the UK [14], Finland [15], the World Health Organization (WHO) [16], and the United States [17, 18].) Many global and national scientific, professional associations, and patient advocacy organisations (including The Obesity Society, Obesity Medicine Association and Obesity Action Coalition in the United States) also consider it a chronic disease [19].

These definitions mark a shift away from viewing obesity as a matter of personal responsibility. They emphasize dynamic interactions between genetic, biological, behavioral, and environmental factors and raise questions about whether existing “obesity prevention” programs truly address the prevention of the disease itself (i.e., prevent the onset of obesity, or slow or stop obesity progression in terms of disease severity and/or development of co-existing conditions) or merely influence broader determinants of health (i.e., health promotion).

This shift is supported by evidence that the relationship between obesity and health behaviors is bi-directional [20], with behaviors often arising from underlying genetic, biological and brain-based pathways [21] that predispose people to obesity as much as they contribute to its development and progression.

### Health Promotion and Broader Determinants of Health

As with all chronic diseases, addressing obesity requires coordinated strategies across health promotion, prevention, and treatment that move beyond only targeting individual behaviors toward upstream systems and downstream clinical treatments.

Understanding obesity as a chronic disease highlights the need to reassess how prevention operates in practice. Interventions commonly labelled “obesity prevention” target individual behavior change via population-level measures designed to address social, economic and environmental determinants of health (i.e., factors beyond genetics and biology, such as income and social status, commercial determinants of health, access to healthcare, literacy, food and physical environments, racism and exposure to racial discrimination, gender bias and exposure to gender-based discrimination, and others) [22].

Fiscal and regulatory interventions provide some of the strongest evidence of impact of policies influencing individual health behavior change and industry practices. Mexico’s 2014 sugar-sweetened beverage tax reduced purchases of taxed drinks by 6%-10% within two years, with larger declines among people living in households with lower income [23, 24]. Similarly, Chile’s front-of-pack labeling and marketing restrictions led to a 24% reduction in purchases of high-sugar beverages [25, 26]. These interventions demonstrate that well-designed policy can shift consumer behavior (reducing purchasing of high-sugar beverages) and reshape industry practices (industries reduced sugar content across many products). However, their effects on long-term obesity prevalence outcomes have not yet been established, underscoring a critical gap between improving determinants of health and preventing a chronic disease driven by complex biological and systemic forces. In the United Kingdom, for instance, the Soft Drinks Industry Levy has resulted in large-scale reformulation and significant reductions in sugar sold in beverages. Effects on obesity prevention have not yet been confirmed by empirical studies and remain based on projection models that assume a causal pathway from policy to reformulation, reduced sugar intake, lower calorie intake, reduced body weight, and reduced obesity risk [27].

### The Limits and Challenges of Obesity Prevention Programs that Focus Only on Individual Health Behaviors

While behavioral interventions often improve eating habits, energy intake and physical activity outcomes, their impact on primary prevention of obesity (i.e., reducing the number of people that develop obesity or secondary prevention which aims to prevent further progression of obesity) remains limited. A recent umbrella review by Jacobs et al. (2025) found that obesity prevention interventions across age groups typically produce only small changes in BMI or BMI z score at the population level, and that only 9 of 60 identified intervention components were rated as “likely effective”, with most others showing limited or inconsistent evidence [13]. The pathway from behavior change to prevention of dysfunctional adiposity and health-related impairments is influenced by many factors beyond individual control, including genetics, early-life nutrition, metabolic regulation and brain-based physiological mechanisms [14, 15].

Thus, interventions that primarily promote healthier behaviors in isolation have not yielded measurable outcomes for obesity prevention. Labeling these initiatives as obesity prevention interventions merely shifts responsibility from upstream policy action changing systems to a downstream focus on changing individual behaviors. This framing frees governments to avoid addressing upstream policy levers, such as food systems, labor conditions, urban design, economic inequality and overarching social determinants of health, that determine exposure to health risks [16]. It also reinforces unrealistic expectations by evaluating upstream health-promoting initiatives solely on their impacts on weight-based outcomes such as BMI, undervaluing their broader benefits for population health and well-being [13].

The complexity of obesity necessitates meaningful primary and secondary prevention that extends well beyond health promotion interventions focused on individual behaviors. Continued framing of obesity through personal responsibility not only limits intervention effectiveness but also reinforces stigma by implying that people with obesity lack discipline or motivation, or that they do not engage in health behaviors. At the same time, mislabeling upstream health promotion initiatives as “obesity prevention” strategies may undermine their value for promoting health, because evaluating them primarily through weight, BMI or obesity outcomes (measures that have shown limited responsiveness [28]) creates the impression that these interventions are ineffective or unnecessary. This signals to policymakers and decision makers that investments in structural and environmental change, as well as interventions aimed to improve health behaviors, do not work, while leaving deeper commercial, social, and economic determinants largely unchallenged. To advance prevention, upstream health promotion programs must be framed for what they are and evaluated on their ability to improve population health, equity, and living conditions, rather than on narrow anthropometric outcomes.

Furthermore, the persistent focus on individual behavior shapes societal narratives that equate body size with personal responsibility and moral worth [29]. This weight-centric narrative, amplified by media and diet culture [30], reinforces stigma and discrimination against people living with obesity [31]. Such stigmatization not only undermines mental and physical health but also deters individuals from seeking appropriate care and contributes to social exclusion and inequity. Public health messaging that focuses narrowly on body weight risks perpetuating harmful stereotypes rather than promoting equitable health outcomes [32, 33].

Increasingly, as explicit weight bias becomes less acceptable, implicit bias remains prevalent and may serve to reinforce a reliance upon behavioral approaches to obesity prevention that have not proven to be effective.

Stigma is therefore not only a social consequence of obesity narratives but also a policy distraction that diverts attention from the structural, economic, and environmental systems that sustain poor health across populations.

### Health-disrupting Environments and Structural Inequities

The social and physical environments that shape daily life drive disease risk by making it easy to engage in unhealthy behaviors and difficult to engage in healthier ones. Aggressive food marketing, long work hours, and limited access to safe spaces for movement interact with people’s biological vulnerability to increase disease risk [34, 35]. Thus, the environments in which we live create constant exposure to triggers that interact with biological and psychological vulnerability to obesity.

However, the burden of these environments is not distributed equally. Socioeconomic disadvantage, discrimination, and geographic isolation restrict access to affordable healthy foods, safe spaces, and healthcare access [36, 37]. Individuals facing these constraints are less able to act on public health recommendations that assume freedom of choice and equal agency [38]. As a result, interventions that aim to modify environments without addressing these social determinants of health risk deepening health disparities rather than reducing them.

Creating genuinely health-promoting environments therefore requires comprehensive actions that target the economic and commercial systems that produce unhealthy food environments and unequal living conditions, through fiscal regulation, labor protections, food system reform, and stronger social policy. Addressing these upstream social determinants of health will not only improve overall health but may also reduce the social pressures that contribute to chronic disease development, progression and persistence.

### Integrating Obesity Prevention and Management

Even in more equitable and health-promoting environments, obesity can arise through biological and metabolic processes that interact with social and environmental factors. Once obesity develops, physiological feedback systems defend higher adiposity levels, making long-term obesity difficult [39].

Genetic evidence further highlights the complexity of obesity. Obesity is highly polygenic, influenced by thousands of genetic variants that each exert small effects, many located in brain regions governing appetite and reward [40]. These findings challenge earlier single-cause explanations such as the “thrifty gene” hypothesis [41] and confirm that genetic predisposition interacts continuously with modern environments. 

Effective obesity prevention therefore requires a truly integrated approach, while acknowledging that there is currently limited empirical evidence on how to design prevention programs that reliably reduce obesity incidence or prevalence. Environmental and behavioral interventions remain essential components of health promotion and chronic disease prevention, but greater emphasis is needed on developing and rigorously evaluating strategies that demonstrate measurable reductions in obesity risk, rather than relying on assumed causal pathways linking macro level changes and policies to health behavior change and subsequent reductions in obesity risk and prevalence. At the same time, person-centered clinical care must be available for individuals already affected by obesity or showing early adiposity related complications, as early identification and treatment can slow disease progression and reduce the risk of serious health consequences [42].

Understanding obesity as a chronic disease highlights the need for coordination across health promotion, prevention, treatment, and management rather than treating these as separate stages. Each level plays a distinct yet complementary role in reducing risk, delaying onset, managing chronic disease, and promoting health and well-being across the continuum of need at the population level.

An integrated framework linking these domains ensures continuity, equity, and efficiency across public health and clinical systems. The World Health Organization (2023) [6] advocates for this unified model, emphasizing coordination between prevention and care to reflect the biological and social realities of living with obesity.

The Irish Health Service Executive Model of Care for Obesity provides an integrated, multi-level approach that links prevention, early intervention, specialist treatment, and long-term management across primary care, community services, and hospital settings, ensuring coordinated support for individuals, families, and populations [43].

### Field Insights and Policy Challenges to Obesity Prevention

Insights from the *Rethinking Obesity Prevention Paradigms* consultation, which involved in-depth interviews with international experts and policymakers working in obesity prevention, reveal persistent barriers shaping current prevention efforts [44].

1. Although obesity is increasingly recognized as a chronic and multifactorial disease, prevention remains fragmented and inconsistently aligned with this understanding. Participants highlighted a lack of consensus on how to define and measure “success” of obesity prevention programs. Most programs still rely on weight or BMI reduction as primary indicators, even though these metrics are crude markers of body size rather than measures of how adipose tissue is negatively affecting a person’s health. This narrow focus limits evaluation of the impact of obesity prevention interventions on health outcomes that matter for population health and for individuals at risk for obesity.
2. Political and funding structures further constrain progress. Short electoral and grant cycles create pressure for rapid, visible results, discouraging sustained, system-level action. Policymakers and practitioners also reported limited authority and resources to implement coordinated, multi-sectoral strategies.
3. Uncertainty about obesity’s classification as a chronic disease continues to undermine integration between public health and clinical programs, including secondary and tertiary prevention, reinforcing outdated lifestyle narratives. Addressing these field-level barriers requires policy frameworks that enable long-term investment, shared accountability, and cross-sector collaboration to align practice with current scientific understanding.
4. Beyond conceptual and measurement challenges, systemic constraints continue to limit the effectiveness of obesity prevention. Short funding and political cycles prioritize quick, visible results over sustainable change, discouraging long-term investment in interventions that address the biological, environmental, and social determinants of obesity. “Short-termism” sustains narrow behavior-focused programs even when evidence calls for deeper systemic reform over the long-term [44].
5. Accountability for prevention remains fragmented across sectors. Health, social, education, and urban planning agencies often pursue disconnected initiatives with limited coordination or shared evaluation frameworks, weakening overall impact. Prevention programs also remain underfunded relative to the scale of obesity as a chronic disease, restricting innovation, evaluation, and workforce capacity.

Structural reform is therefore essential. Longer evaluation windows would enable measurement of gradual but meaningful improvements in obesity risk. Cross-sector partnerships linking health, education, agriculture, and social policy could foster shared accountability and coherent policy design. Consistent classification of obesity as a chronic disease, aligned with WHO guidance [6] would further integrate prevention and treatment within national systems.

Without such changes, prevention efforts will remain fragmented and reactive rather than strategic and sustained. Addressing these barriers is crucial to building a durable, evidence-based foundation for obesity prevention within broader health system reform.

## Conclusion

Redefining obesity as a chronic, multifactorial disease requires a fundamental shift in how obesity prevention is designed and delivered. Most current programs improve environments and health behaviors but do not directly address the biological, physiological, and clinical mechanisms underlying adiposity-related disease. These programs remain valuable for population health outcomes but have limited direct impact on obesity without systemic and clinical alignment.

A coherent strategy must link population-level health promotion, targeted disease prevention, and long-term disease treatment and management within a single, coordinated framework supported by sustained investment and cross-sector accountability. Cross-sectoral collaboration will also be critically important for addressing equity gaps that disproportionally impact marginalized populations, such as racialized and low-income communities. No single sector can dismantle these forces alone.

Policy should embed equity, stigma reduction, and person-centered care at every level. Meaningful progress will depend on shared commitment from researchers, clinicians, policymakers, and people living with obesity to adopt outcomes that reflect real health gains such as metabolic, physical and social functioning, and mental health [45], complication prevention, and enhanced quality of life [46], the latter of which is of primary importance to individuals living with obesity.

## Data Availability

No datasets were generated or analysed during the current study.

## References

[1] Dai H, Alsalhe TA, Chalghaf N, Riccò M, Bragazzi NL, Wu J. The global burden of disease attributable to high body mass index in 195 countries and territories, 1990–2017: An analysis of the Global Burden of Disease Study. PLoS Med United States. 2020;17:e1003198. 10.1371/journal.pmed.1003198. 10.1371/journal.pmed.1003198 PMC7386577 32722671

[2] The 1st International Conference on Health Promotion, Ottawa. 1986. https://www.who.int/teams/health-promotion/enhanced-wellbeing/first-global-conference.

[3] World Health Organization. Healthy cities approach. Europe: World Health Organization. 2026. https://www.who.int/europe/groups/who-european-healthy-cities-network. WHO European Healthy Cities Network.

[4] World Health Organization. Health promotion and disease prevention through population-based interventions, including action to address social determinants and health inequity. https://www.emro.who.int/about-who/public-health-functions/health-promotion-disease-prevention.html.

[5] National Diabetes Prevention Program. National Diabetes Prevention Program. U.S. U.S. Centers for Disease Control and Prevention. https://www.cdc.gov/diabetes-prevention/index.html. Accessed 6 Feb 2026. Cited 6 Feb 2026.

[6] World Health Organization. Health service delivery framework for prevention and management of obesity. World Health Organization. 2023. https://www.who.int/publications/i/item/9789240073234.

[7] Health Services Executive H. HSE Specialist Weight Management Service. HSE weight management support programme. https://www2.hse.ie/conditions/obesity/supports-courses/#hse-weight-management-support-programme. Accessed 6 Feb 2026. Cited 6 Feb 2026.

[8] Wharton S, Lau DCW, Vallis M, Sharma AM, Biertho L, Campbell-Scherer D et al. Obesity in adults: a clinical practice guideline. CMAJ. 2020;192:E875–91. 10.1503/cmaj.191707. Cited 14 Apr 2023. 10.1503/cmaj.191707 PMC7828878 32753461

[9] Breen C, O’Connell J, Geoghegan J, O’Shea D, Birney S, Tully L et al. Obesity in adults: a 2022 adapted clinical practice guideline for Ireland. Obesity Facts. 2022;15:736–52. 10.1159/000527131. Cited 28 Mar 2024. 10.1159/000527131 PMC9801383 36279848

[10] Preiss Contreras Y, Ramos Salas X, Ávila Oliver C, Saquimux Contreras MA, Muñoz Claro R, Canales Ferrada C. Obesity in adults: Clinical practice guideline adapted for Chile. Medwave Chile. 2022;22:e2649. 10.5867/medwave.2022.10.2649. 10.5867/medwave.2022.10.2649 36427185

[11] Socialstyrelsen S. Nationella riktlinjer: obesitas. Stockholm: Socialstyrelsen. 2023. https://www.socialstyrelsen.se/kunskapsstod-och-regler/regler-och-riktlinjer/nationella-riktlinjer/riktlinjer-och-utvarderingar/obesitas/. Accessed 20 Feb 2026. Cited 20 Feb 2026.

[12] Partnership Overweight Netherlands. Overweight and obesity in adults and children. Overgewicht en obesitas bij volwassenen en kinderen. 2023. https://richtlijnendatabase.nl/richtlijn/overgewicht_en_obesitas_bij_volwassenen_en_kinderen/startpagina_richtlijn_overgewicht_en_obesitas_bij_volwassenen_en_kinderen.html. Accessed 8 Jan 2025. Cited 8 Jan 2025.

[13] Chávez-Manzanera EA, Vera-Zertuche JM, Kaufer-Horwitz M, Vázquez-Velázquez V, Flores-Lázaro JR, Mireles-Zavala L, et al. Mexican Clinical Practice Guidelines for Adult Overweight and Obesity Management. Curr Obes Rep United States. 2024;13:643–66. 10.1007/s13679-024-00585-w. 10.1007/s13679-024-00585-w PMC11522083 39356455

[14] NICE NI for H and CE. Overweight and obesity management NICE Guideline. UK: National Institute for Health and Care Excellence. 2025. https://www.nice.org.uk/guidance/ng246. Accessed 20 Feb 2026. Cited 20 Feb 2026.

[15] Obesity (children, adolescents and adults); Current care recommendation. Käypä hoito - Current care guidelines. Finland: a working group set up by the Finnish Medical Association Duodecim, the Finnish Obesity Research Association and the Finnish Pediatric Association. 2025. https://www.kaypahoito.fi/hoi50124. Accessed 20 Feb 2026. Cited 20 Feb 2026.

[16] Celletti F, Farrar J, De Regil L. World Health Organization Guideline on the Use and Indications of Glucagon-Like Peptide-1 Therapies for the Treatment of Obesity in Adults. JAMA United States. 2026;335:434–8. 10.1001/jama.2025.24288. 10.1001/jama.2025.24288 41324410

[17] Hampl SE, Hassink SG, Skinner AC, Armstrong SC, Barlow SE, Bolling CF, et al. Clinical Practice Guideline for the Evaluation and Treatment of Children and Adolescents With Obesity. Pediatr United States. 2023;151:e2022060640. 10.1542/peds.2022-060640. 10.1542/peds.2022-060640 36622115

[18] Bannuru RR. Introduction and methodology: Standards of Care in Overweight and Obesity–2025. BMJ Open Diabetes Res Care [Internet]. 2025;13:e004928. 10.1136/bmjdrc-2025-004928. 40360275 10.1136/bmjdrc-2025-004928

[19] IOC IOC. International obesity collaborative consensus statement. The Obesity Society. 2024. https://www.obesity.org/international-obesity-collaborative/. Accessed 20 Feb 2026. Cited 20 Feb 2026.

[20] Parent MB, Higgs S, Cheke LG, Kanoski SE. Memory and eating: A bidirectional relationship implicated in obesity. Neurosci Biobehavioral Reviews [Internet]. 2022;132:110–29. 10.1016/j.neubiorev.2021.10.051. 10.1016/j.neubiorev.2021.10.051 PMC8816841 34813827

[21] Saeed S, Bonnefond A, Froguel P. Obesity: exploring its connection to brain function through genetic and genomic perspectives. Mol Psychiatry [Internet]. 2025;30:651–8. 10.1038/s41380-024-02737-9. 39237720 10.1038/s41380-024-02737-9 PMC11746128

[22] Ramos Salas X, Forhan M, Caulfield T, Sharma AM, Raine K. A critical analysis of obesity prevention policies and strategies. Can J Public Health [Internet]. 2017;108:e598–608. 10.17269/CJPH.108.6044. [cited 2023 Apr 14];. 31823280 10.17269/CJPH.108.6044 PMC6972457

[23] Colchero MA, Rivera-Dommarco J, Popkin BM, Ng SW. Mexico, Evidence Of Sustained Consumer Response Two Years After Implementing A Sugar-Sweetened Beverage Tax. Health Aff (Millwood). United States. 2017;36:564–71. 10.1377/hlthaff.2016.1231. 10.1377/hlthaff.2016.1231 PMC5442881 28228484

[24] Colchero MA, Popkin BM, Rivera JA, Ng SW. Beverage purchases from stores in Mexico under the excise tax on sugar sweetened beverages: observational study. BMJ [Internet]. 2016;352:h6704. 10.1136/bmj.h6704. 26738745 10.1136/bmj.h6704 PMC4986313

[25] Taillie LS, Reyes M, Colchero MA, Popkin B, Corvalán C. An evaluation of Chile’s Law of Food Labeling and Advertising on sugar-sweetened beverage purchases from 2015 to 2017: A before-and-after study. PLoS Med United States. 2020;17:e1003015. 10.1371/journal.pmed.1003015. 10.1371/journal.pmed.1003015 PMC7012389 32045424

[26] CIAPEC. 7th anniversary of the Food Labeling and Advertising Law. 7 successful results and opportunities to enhance them. Santiago, Chile: Center for Research in Food Environments and Prevention of Chronic Diseases Associated with Nutrition - CIAPEC; https://ciapec.cl/en/noticias/7o-aniversario-de-la-ley-de-etiquetado-y-publicidad-de-los-alimentos-7-resultados-exitosos-y-oportunidades-para-potenciarlos/.

[27] Cobiac LJ, Law C, Smith R, Cummins S, Rutter H, Rayner M, et al. Population health and health sector cost impacts of the UK Soft Drinks Industry Levy: a modelling study. Public Health Res (Southampt) Engl. 2025;1–17. 10.3310/GJMW1501. 10.3310/GJMW1501 41351382

[28] Swinburn BA, Kraak VI, Allender S, Atkins VJ, Baker PI, Bogard JR et al. The Global Syndemic of Obesity, Undernutrition, and Climate Change: The Lancet Commission report. The Lancet. Elsevier. 2019;393:791–846. 10.1016/S0140-6736(18)32822-8. Cited 5 Nov 2025. 10.1016/S0140-6736(18)32822-8 30700377

[29] Ramos Salas X. The ineffectiveness and unintended consequences of the public health war on obesity. Can J Public Health Switz. 2015;106:e79–81. 10.17269/cjph.106.4757. 10.17269/cjph.106.4757 25955676

[30] Hogan L, Johnson KJ, Faw MH. Diet culture beliefs, co-rumination, intuitive eating, and experiences of internalized weight stigma in the adjustment to college among emerging adult women. Emerging Adulthood. SAGE Publications Inc; 2025;13:833–44. 10.1177/21676968251338573. Cited Sept 2025.

[31] Westbury S, Oyebode O, van Rens T, Barber TM. Obesity Stigma: Causes, Consequences, and Potential Solutions. Curr Obes Rep United States. 2023;12:10–23. 10.1007/s13679-023-00495-3. 10.1007/s13679-023-00495-3 PMC9985585 36781624

[32] Nutter S, Eggerichs LA, Nagpal TS, Ramos Salas X, Chin Chea C, Saiful S, et al. Changing the global obesity narrative to recognize and reduce weight stigma: A position statement from the World Obesity Federation. Obes Reviews [Internet]. 2024;25:e13642. 10.1111/obr.13642. 10.1111/obr.13642 PMC13019846 37846179

[33] Robinson M, Chenery M, Smith J. Beyond Choice: Challenging Individualism in Public Health Narratives. Health Promot J Austr Australia. 2026;37:e70125. 10.1002/hpja.70125. 10.1002/hpja.70125 41152130

[34] Boyland E, Muc M, Coates A, Ells L, Halford JCG, Hill Z, et al. Food marketing, eating and health outcomes in children and adults: a systematic review and meta-analysis. Br J Nutr Engl. 2025;133:781–805. 10.1017/S0007114524000102. 10.1017/S0007114524000102 PMC12169957 40518855

[35] Boyland E, Backholer K, Potvin Kent M, Bragg MA, Sing F, Karupaiah T, et al. Unhealthy Food and Beverage Marketing to Children in the Digital Age: Global Research and Policy Challenges and Priorities. Annu Rev Nutr United States. 2024;44:471–97. 10.1146/annurev-nutr-062322-014102. 10.1146/annurev-nutr-062322-014102 38631811

[36] Løvhaug AL, Granheim SI, Djojosoeparto SK, Harrington JM, Kamphuis CBM, Poelman MP, et al. The potential of food environment policies to reduce socioeconomic inequalities in diets and to improve healthy diets among lower socioeconomic groups: an umbrella review. BMC Public Health Engl. 2022;22:433. 10.1186/s12889-022-12827-4. 10.1186/s12889-022-12827-4 PMC8895543 35246074

[37] Carrillo-Alvarez E, Rifà-Ros R, Salinas-Roca B, Costa-Tutusaus L, Lamas M, Rodriguez-Monforte M. Diet-Related Health Inequalities in High-Income Countries: A Scoping Review of Observational Studies. Adv Nutr United States. 2025;16:100439. 10.1016/j.advnut.2025.100439. 10.1016/j.advnut.2025.100439 PMC12149430 40334986

[38] Clouston SAP, Link BG. A retrospective on fundamental cause theory: State of the literature, and goals for the future. Annu Rev Sociol United States. 2021;47:131–56. 10.1146/annurev-soc-090320-094912. 10.1146/annurev-soc-090320-094912 PMC8691558 34949900

[39] Sumithran P, Proietto J. The defence of body weight: a physiological basis for weight regain after weight loss. Clin Sci (Lond) Engl. 2013;124:231–41. 10.1042/CS20120223. 10.1042/CS20120223 23126426

[40] Loos RJF, Yeo GSH. The genetics of obesity: from discovery to biology. Nat Rev Genet Engl. 2022;23:120–33. 10.1038/s41576-021-00414-z. 10.1038/s41576-021-00414-z PMC8459824 34556834

[41] Speakman JR. Evolutionary perspectives on the obesity epidemic: adaptive, maladaptive, and neutral viewpoints. Annu Rev Nutr United States. 2013;33:289–317. 10.1146/annurev-nutr-071811-150711. 10.1146/annurev-nutr-071811-150711 23862645

[42] Busetto L, Dicker D, Frühbeck G, Halford JCG, Sbraccia P, Yumuk V, et al. A new framework for the diagnosis, staging and management of obesity in adults. Nat Med [Internet]. 2024. 10.1038/s41591-024-03095-3. 38969880 10.1038/s41591-024-03095-3

[43] Health Services Executive. HSE model of care for overweight and obesity management. Ireland: HSE Ireland. https://easo.org/video/hse-model-of-care/. Accessed 20 Feb 2026. Cited 20 Feb 2026.

[44] Ramos Salas X, Hussey B. Rethinking obesity prevention paradigms: an expert consultation. Replica Communications. https://replicagroup.com/site/assets/Report_Rethinking_Obesity_Prevention_Paradigms_final.pdf.

[45] Sharma AMM, M M. & M: a mnemonic for assessing obesity. Obesity Reviews. Wiley, Ltd. 2010;11:808–9. 10.1111/j.1467-789X.2010.00766.x. Cited 23 Feb 2026. 10.1111/j.1467-789X.2010.00766.x 21182728

[46] Dijkhorst PJ, Monpellier VM, Terwee CB, Liem RSL, van Wagensveld BA, Janssen IMC, et al. Core Set of Patient-Reported Outcome Measures for Measuring Quality of Life in Clinical Obesity Care. Obes Surg United States. 2024;34:2980–90. 10.1007/s11695-024-07381-4. 10.1007/s11695-024-07381-4 PMC11289183 39008218

